# Recruitment capacity and operational barriers to prehospital cardiac electrical biomarker acquisition: an ambulance-based pilot study

**DOI:** 10.1186/s12872-026-06145-5

**Published:** 2026-06-26

**Authors:** Aurèle Nicolet, Laurent Suppan, Loric Stuby

**Affiliations:** 1 Ambulance Région Bienne, Emergency Medical Services, Bienne, Switzerland; 2 Division of Emergency Medicine, Department of acute care medicine, Geneva University Hospitals, Geneva, Switzerland; 3 Department of anaesthesiology, pharmacology, intensive care and emergency medicine, Faculty of Medicine, University of Geneva, Geneva, Switzerland; 4 Genève TEAM Ambulances, Emergency Medical Services, Geneva, Switzerland

**Keywords:** Cardiac electrical biomarker, Myocardial infarction, Prehospital, Diagnosis, Ambulance, Emergency medical services, Detection

## Abstract

**Background:**

In prehospital settings, myocardial infarction (MI) diagnosis relies on symptoms and electrocardiogram (ECG) analysis. The cardiac electrical biomarker (CEB) has been developed to quantify abnormal multipolar electrical activity through a 12-lead ECG, reflecting ischemia-related cellular disruption. This study primarily aimed to assess recruitment capacity and operational feasibility for a future large-scale prospective prehospital study involving CEB measurements. Secondary objectives were to describe successful CEB acquisition and to identify practical barriers to data collection in the prehospital environment.

**Methods:**

This pilot study was a secondary analysis of prospectively collected observational data from the “Ambulance Région de Bienne” service (covering 120,000 inhabitants) between March and September 2023. Adult patients managed for suspected acute coronary syndrome who underwent a prehospital 12-lead ECG with attempted CEB acquisition and were subsequently admitted to the Biel Hospital Centre were eligible. Analyses were descriptive and focused on feasibility-related endpoints.

**Results:**

A total of 70 patients were identified, of whom 60 met eligibility criteria. Regarding recruitment feasibility, a mean enrolment rate of 7.7 patients per month was observed, corresponding to roughly one patient per 70 prehospital missions. Regarding operational experience with CEB acquisition, technical and practical barriers were identified, including telemetry connectivity issues, workflow constraints, and documentation limitations. Successful CEB acquisition was variable; however, the true failure rate could not be reliably determined due to incomplete systematic documentation of all attempted acquisitions.

**Conclusions:**

Recruitment of eligible patients for a future large-scale study appears potentially feasible, with an observed rate of 7.7 patients per month within this single EMS. However, while operational barriers were identified, reliable CEB acquisition could not be assessed due to incomplete capture of failed acquisition attempts. These findings primarily inform study design and implementation strategy for future investigations.

## Introduction

### Background

Acute coronary syndromes represent a significant challenge in Swiss prehospital care, with nearly 20,000 myocardial infarctions occurring annually [1]. The Swiss prehospital system has traditionally relied on symptom assessment and electrocardiogram (ECG) analysis, both of which are subject to limitations, particularly in non-occlusive myocardial infarction (NOMI) or non-ST-elevation presentation. While troponin measurement remains the gold standard for myocardial infarction diagnosis in hospital settings, its processing time often exceeds typical transport times in Swiss urban areas, limiting its prehospital utility in this setting at present [2–5]. Furthermore, the dynamic prehospital environment presents unique challenges for diagnostic accuracy not encountered in hospital settings.

The cardiac electrical biomarker (CEB) is an algorithm-derived parameter calculated from enhanced multipolar electrical activity recorded from the precordial lead V2, designed to quantify the probability of acute myocardial injury beyond conventional visual ECG interpretation. The concept underlying the CEB was first investigated in earlier research exploring electrical signatures of acute myocardial injury. A 2014 study suggested that CEB-derived indices could be more effective in identifying acute ischemic injury than visual ECG interpretation by emergency physicians or cardiologists [6]. Subsequent research conducted in emergency department settings demonstrated a positive correlation between high-sensitivity troponin levels and CEB values, indicating that CEB may reflect underlying myocardial damage [7]. In 2018, a prospective study at the University Hospital of Basel reported that combining CEB with visual ECG interpretation increased sensitivity from 43% to 79% for detecting non-ST-elevation myocardial infarction (NSTEMI) [8], but its impact on hard clinical outcomes could not be established. A 2019 study evaluating a Vectraplex electrocardiogram system incorporating CEB assessment in patients with suspected ST-elevation myocardial infarction reported moderate sensitivity and specificity, further highlighting the need for continued evaluation of this technology across different clinical contexts [9].

In 2020, the technology was further developed and integrated into Corpuls^®^3 monitors by VectraCor Inc. (New Jersey, USA), enabling potential application in clinical and prehospital settings. Before evaluating the diagnostic performance of CEB in the prehospital setting, it is necessary to determine whether reliable data acquisition is feasible under routine prehospital conditions. This pilot study therefore exclusively focused on feasibility outcomes to inform the design of a future prospective trial.

### Objectives

This study primarily aimed to assess recruitment capacity and operational feasibility for a future large-scale prospective prehospital study involving CEB measurements in patients 18 years or older who benefitted from an ECG acquisition in the context of a suspected acute coronary syndrome. Secondary objectives were to describe successful CEB acquisition and to identify practical barriers to data collection in the prehospital environment.

## Methods

### Study design and period

This pilot study was a secondary analysis of prospectively collected observational data from a single emergency medical service (EMS): “Ambulance Région de Bienne (ARB)”. CEB acquisition was prospectively attempted during routine patient care according to a standardized operating procedure between March 1st and September 30th, 2023, as part of a paramedic diploma thesis, whereas the present feasibility analysis was conducted retrospectively after completion of the study period.

This study was designed as a pilot feasibility study prior to conducting a larger prospective diagnostic accuracy trial. Conducting feasibility assessments before full-scale diagnostic evaluations is recommended to identify operational barriers, recruitment capacity, and data collection constraints, particularly in complex prehospital environments [10–12]. The present methodology therefore focused on recruitment rate, operational constraints, and completeness of data acquisition rather than diagnostic performance metrics.

### Study setting

In 2023, the ARB EMS served a population of 122,708, operating 4 to 5 ambulances during the day and 3 at night. EMS teams typically consisted of 2 to 3 members, including at least one paramedic. Some paramedics held an additional nurse anaesthetist qualification. Teams could also include emergency medical technicians and paramedic trainees. Over the year, this EMS responded to a total of 7,887 missions, comprising 6,613 primary interventions (unscheduled emergency responses to patients at the scene) and 1,274 secondary interventions (such as interhospital or facility transfers). Notably, 3,789 of the primary interventions occurred during the defined study period. All operational ambulance units and all clinical staff working during the study period were able to enrol. Recruitment was therefore possible 24 h a day, 7 days a week. A standardized operating procedure for CEB acquisition was shared with the entire service prior to study initiation, and no restrictions were placed on shifts, days, or individual professionals.

### Study population

Adult patients aged 18 years or older who were managed for suspected acute coronary syndrome and in whom CEB acquisition was attempted during routine care were eligible. Eligibility was based on attempted acquisition and was not conditional on successful CEB measurement. Patients were included in the final analysis if they were subsequently admitted to the Biel Hospital Centre and had a medical diagnosis documented during their emergency department stay.

The following exclusion criteria applied:

- Age < 18 years;
- Missing data (CEB values, medical diagnosis, or ECG);
- ECG not usable, defined by at least one of the following:
  - ⚬ Missing lead(s);
  - ⚬ Excessive noise preventing reliable ECG interpretation (P, QRS, and T waves had to be identifiable);
  - ⚬ ST-segment baseline deviation greater than 5 mm.

Patients with missing or non-interpretable CEB data were excluded from the final analysis of the original diploma thesis because no analysable measurements were available. These exclusion criteria were predefined in the original diploma thesis protocol.

Importantly, the study database only contained patients for whom a CEB acquisition attempt was documented. Consequently, the total number of undocumented acquisition attempts could not be determined retrospectively. Patients excluded due to missing leads or non-interpretable ECGs were considered technically unsuccessful acquisitions among documented attempts. This limitation was taken into account in the interpretation of feasibility outcomes and is further discussed in the limitations section.

### CEB definition and thresholds

The CEB values are derived from a quantification of multipolar cardiac electrical activity occurring during myocardial injury, computed automatically from a standard 12-lead ECG. This measurement is performed by the Corpuls^®^3 monitor using a proprietary algorithm developed by VectraCor Inc. that integrates electrical signals across all leads and compares them to normal dipolar activity. Results are obtained within one minute after performing the ECG.

Pathophysiologically, during myocardial ischemia, insufficient oxygen for cellular energy production forces cells to produce adenosine triphosphate anaerobically, increasing lactate levels and intracellular protons. This acidosis alters membrane permeability, disrupting ionic balance and electrical polarization [13]. The resulting potential gradients between healthy and affected cells create dipole foci and local electric fields, adding to the depolarization wave and forming multipolar electrical activity [7]. CEB quantifies this abnormal activity relative to normal dipolar activity to estimate the probability of acute ischemic myocardial injury [8]. VectraCor Inc. has established the following CEB value thresholds:

- 1–64: normal dipolar electrical heart activity;
- 65–94: undetermined;
- > 94: clear indication suggesting acute myocardial injury.

Accurate CEB calculation requires correct electrode placement (particularly V2), bidirectional telemetry transmission, and automatic analysis by the proprietary software. Due to its proprietary nature, the detailed algorithm is not publicly disclosed. Interpretation relies on this automated, device-specific measurement, and values obtained outside this system may not be equivalent.

### CEB acquisition procedure

The CEB acquisition procedure was implemented at the EMS from March 1st to September 30th, 2023. To obtain a CEB value, personnel followed a standardized step-by-step procedure:

- 1) Verifying the correct configuration between the team and the ambulance’s dedicated Corpuls^®^3 monitor;
- 2) Obtaining a 12-lead ECG in patients suspected of having acute coronary syndrome following internal protocols while ensuring the correct position of the V2 lead electrode (the lead used to compute CEB);
- 3) Sending the ECG via telemetry, regardless of its appearance, as this is required to obtain the CEB value;
- 4) Clicking the “ECGmax” button to obtain the printed ECG with the CEB value displayed.

Additionally, EMS personnel completed a case report form in the EMS office with the ID number, enabling linkage between the ECGmax and the final ED diagnosis. This linkage was facilitated through authorized access to the hospital patient database software (KISIM) granted to the first author (Aurèle Nicolet) for a limited time by the Biel Hospital Centre information technology department for his diploma thesis. Only the final ED diagnosis was extracted from KISIM, and intervention ID numbers were systematically deleted from the dataset before anonymous sharing with coauthors. Patient care was not influenced by the study.

### Outcomes

The primary outcome was the recruitment rate, defined as the number of eligible patients with attempted CEB acquisition per month. It was selected as the primary feasibility outcome because it directly reflects the operational capacity to enrol eligible patients in the prehospital environment, which is a key determinant of feasibility for a larger prospective study.

Secondary outcomes included the documented successful CEB acquisitions and exploratory identification of practical barriers to acquisition. In cases of unsuccessful or incomplete CEB acquisition, potential barriers were explored through informal, non-systematic, individual discussions with the EMS personnel involved. Identified barriers were summarized descriptively and were intended to provide insights for feasibility, rather than to serve as systematically measured outcomes.

### Statistical analysis

Analyses were descriptive and focused exclusively on feasibility outcomes. Continuous variables are reported as medians with ranges where appropriate, and categorical variables as counts and proportions. No hypothesis testing or diagnostic accuracy analyses were performed. Statistical analyses were performed using Stata Statistical Software: Release 15.1 (StataCorp LLC, College Station, TX, USA).

Due to the pilot study design primarily assessing recruitment rate and feasibility, no prior sample size calculation was performed. The present feasibility analysis was conducted retrospectively after completion of the study period; a post-hoc sample size calculation was therefore not performed, as it would not reflect the original pilot design and would amount to retrospective rationalization rather than genuine sample size planning.

## Results

**Fig. 1:**
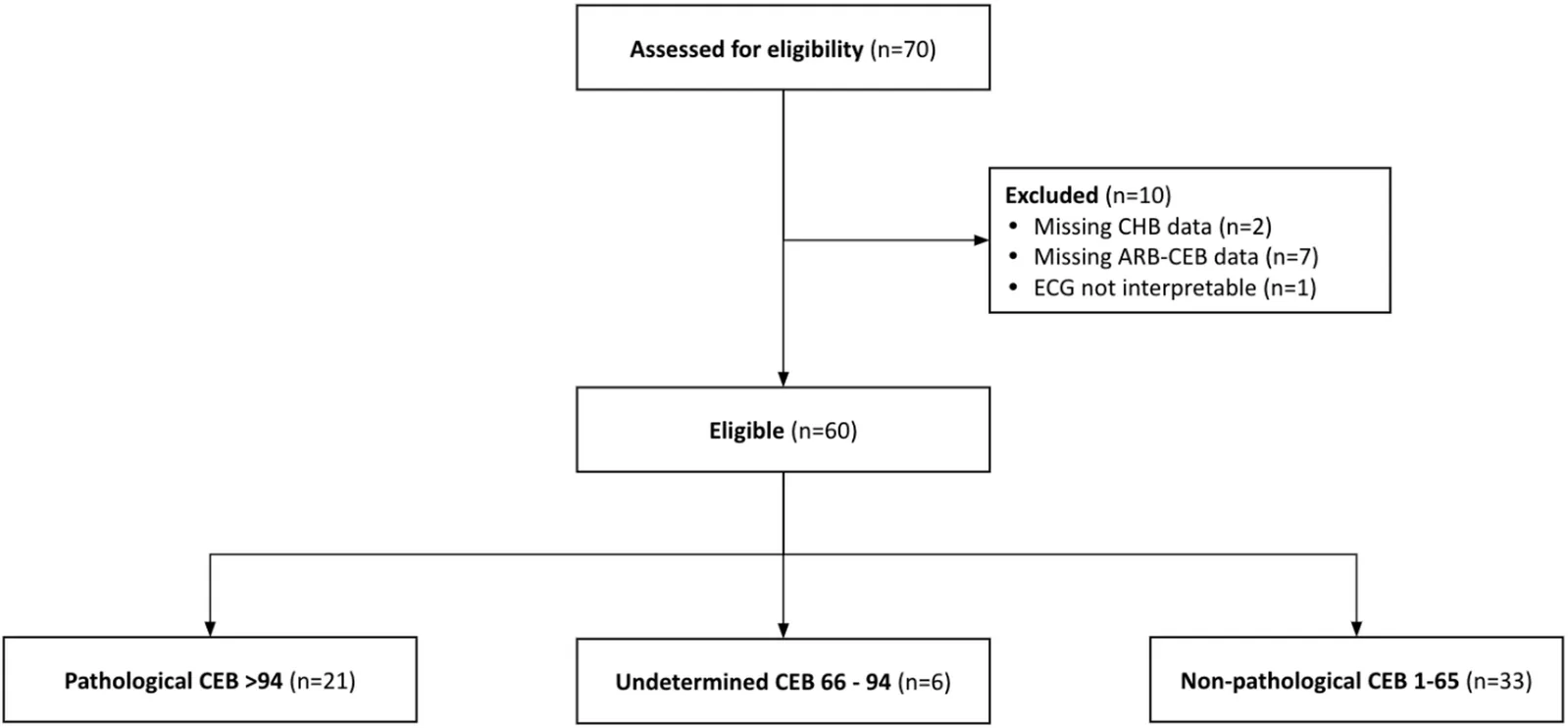
Flowchart. CHB = Biel Hospital Centre, ARB = Ambulance Région Bienne, CEB = Cardiac Electrical Biomarker, ECG = electrocardiogram. CEB result categories (pathological, indeterminate, non-pathological) are based on manufacturer-defined thresholds (VectraCor Inc.) as described in Sect.  2.4, and are reported descriptively to inform the design of a future diagnostic accuracy study. There was an unknown number of additional undocumented acquisition attempts, which could not be retrieved retrospectively

A total of 70 patients were identified, of whom 60 met the eligibility criteria (Fig. 1). The study population age ranged from 26 to 96 years (median: 76 years). Men represented 55% (*n* = 33) of subjects and women 45% (*n* = 27).

### Recruitment rate

During the 7-month study period, the initial recruitment rate was 10 patients per month. This rate decreased to 8.6 patients per month when accounting for retrospectively excluded patients, and further dropped to 7.7 patients per month after excluding cases with undetermined CEB values. This additional stratification was predefined in the original diploma thesis and is reported here to provide a pragmatic estimate of the recruitment rate of fully interpretable CEB values, as this parameter is directly relevant for sample size planning in a future diagnostic accuracy study, where indeterminate CEB values would not contribute to sensitivity or specificity estimates.

During the study period, 3,789 primary interventions were performed, of which 54 yielded usable CEB values, corresponding to an enrolment rate of 1.4% (54/3,789), i.e., approximately one inclusion per 70 interventions.

### Operational experience with CEB acquisition

Of the 70 patients initially identified, 7 (10%) had no CEB value due to telemetry failure preventing ECGmax acquisition. In addition, an unknown number of additional attempted acquisitions were never documented and could not be retrieved retrospectively. As a result, the total number of unsuccessful acquisition attempts could not be determined, and no precise overall failure rate can be reported. Among the documented failures (*n* = 7), all were attributed to telemetry connectivity issues. Other potential barriers were identified qualitatively but could not be quantified due to the absence of standardized and systematic data collection.

### Qualitative observational impressions of practical barriers to acquisition

During the study period, multiple practical challenges arose that hindered the successful acquisition of CEB measurements. The following observations are based on informal, non-systematic feedback from EMS personnel and should be interpreted as qualitative impressions rather than systematically analysed findings. No implementation science framework was applied, and no hierarchy of frequency or importance can be established from these data:

- Telemetry connectivity issues: failed bidirectional ECG transmission between the ambulance unit and the remote server was the most frequent cause of missing or indeterminate CEB values. Network interruptions or poor signal quality directly limited data acquisition.
- Operational workflow constraints: the dynamic prehospital environment, including time-sensitive patient care and variable EMS team composition, occasionally limited opportunities to perform CEB measurements according to protocol.
- Data documentation limitations: early study procedures relied on paper-based recording of attempted CEB acquisitions, which made it difficult to systematically track failed attempts. Transition to fully electronic patient care records should improve traceability in future studies.

## Discussion

This ambulance-based pilot study was designed to assess recruitment capacity, workflow integration, and operational barriers associated with prehospital CEB acquisition, and to inform the design of a future large-scale prospective study. The main findings indicate that recruitment and technical acquisition feasibility represent two distinct dimensions of this pilot study: while patient recruitment within a single EMS appeared feasible and predictable, substantial technical and procedural challenges currently limit reliable data acquisition. Importantly, this study should be interpreted as an operational pilot focusing on recruitment capacity and system-level constraints rather than an evaluation of CEB technology performance.

### Recruitment capacity in the prehospital setting

Feasibility studies are intended to determine whether key processes required for a future definitive trial can be successfully implemented, including recruitment capability, data acquisition reliability, and operational integration within routine care [14]. Methodological guidance for pilot and feasibility studies emphasizes that such evaluations are essential before undertaking larger effectiveness or diagnostic accuracy trials, particularly in complex healthcare environments [10, 15].

The observed recruitment rate of 7.7 eligible patients per month suggests that enrolment of patients with suspected acute coronary syndrome is achievable within routine prehospital practice. This rate is consistent with the operational realities of EMS, where only a subset of patients presenting with chest pain or related symptoms undergo a 12-lead ECG. Although recruitment was not consecutive and some degree of selection bias cannot be excluded, the results provide a preliminary estimate of enrolment capacity that may inform planning of a future study. Importantly, recruitment feasibility must be interpreted in the context of the study’s pragmatic design. Patient inclusion was embedded in routine clinical care and did not rely on the presence of a study investigator, supporting the external validity of the recruitment estimates. Scaling this design to multiple EMS systems may allow adequate enrolment within a reasonable timeframe, provided that technical barriers to data acquisition are addressed. However, the recruitment rate observed in this study is likely context-dependent, reflecting the specific operational characteristics, patient volume, and EMS team behaviour of a single service, and may not be directly transferable to other settings.

### Operational experience with CEB acquisition

Conducting research in the prehospital setting presents well-described system-level constraints and methodological challenges, including environmental variability, time pressure, infrastructure dependency, and limited control over technical conditions [16–18].

The technical barriers identified in this study are consistent with these structural characteristics of prehospital research and underscore the importance of context-specific feasibility assessment prior to evaluating diagnostic performance.

While recruitment proved feasible, successful acquisition of usable CEB data was inconsistent. The most prominent barrier identified was the dependency on telemetry-based ECG transmission, which frequently failed due to network connectivity issues. As CEB calculation required successful bidirectional data transfer between the ambulance and a remote server, connectivity limitations directly translated into missing data. This finding has important implications for future study design. First, systematic documentation of all attempted CEB acquisitions including failures is essential to accurately quantify acquisition success rates and identify context-specific determinants of failure. Second, feasibility of CEB acquisition is closely tied to local network infrastructure and technical configuration, meaning that results from one EMS system may not be directly generalizable without accounting for these factors.

Beyond network connectivity, procedural aspects such as ECG electrode placement, particularly correct positioning of the V2 lead, represent additional sources of variability. Although ECG acquisition is a routine skill for paramedics, small deviations in electrode placement may have disproportionate effects on derived CEB. This highlights the need for targeted training, standardized verification procedures, and possibly redundancy measures (e.g. repeated recordings) in future studies.

### Implications for future study design

This pilot study provides several actionable insights for the design of a future prospective trial. First, technical prerequisites including network reliability, data storage, and traceability of failed acquisitions must be optimized prior to expanding enrolment. Secondly, study protocols should explicitly account for the dynamic and heterogeneous nature of the prehospital environment, which differs fundamentally from controlled hospital-based research settings. Importantly, while recruitment feasibility was assessed, the reliability of obtaining analysable CEB measurements under real-world prehospital conditions could not be fully determined in this study. In accordance with recommendations for staged clinical research development, these feasibility data provide essential parameters for designing a subsequent prospective diagnostic accuracy study, including anticipated recruitment rates, expected data loss, and required technical adaptations [14].

### Limitations

This study was monocentric and exploratory in nature. Although patients were intended to be included consecutively, complete verification of consecutive inclusion was not possible, and therefore a selection bias cannot be ruled out. In addition, the inability to systematically capture all CEB acquisition attempts precludes reliable quantification of the acquisition failure rate. This represents a non-recoverable structural limitation inherent to the retrospective design, rather than a reporting limitation addressable through improved documentation alone. As a consequence, neither failure rate estimation nor comparative feasibility assessment across EMS systems can be supported by these data, even for descriptive purposes. In addition, exclusion of patients with failed or missing CEB acquisition may have introduced analysis-stage selection bias, as technical infeasibility itself limited evaluable cases. Feasibility benchmarks were not prespecified, and data collection relied on routine clinical practice, limiting control over technical and operational variables. These limitations are inherent to pilot feasibility studies and directly inform the design requirements for future prospective investigations. Additionally, these results reflect settings in well-resourced, high-income countries with efficient EMS systems and robust network infrastructure. The development and deployment of technologies like CEB predominantly in such contexts risk widening healthcare inequalities between wealthy and low-resource countries. Future research should consider these disparities to promote more equitable diagnostic solutions globally.

## Conclusions

This pilot study suggests that recruitment of patients with suspected acute coronary syndrome is feasible in a prehospital EMS setting, with an estimated enrolment rate of 7.7 patients per month within this single EMS. This finding is consistent with the operational viability of a future larger-scale prospective study. Several operational and technical barriers to implementation of CEB measurements were identified, including telemetry connectivity issues, workflow constraints in the prehospital environment, and limitations in data documentation processes. These findings provide important information for optimizing study logistics and staff training in future prospective studies. However, the extent of reliable CEB acquisition could not be determined in this study, as the systematic capture of all attempted acquisitions and failures was incomplete. Consequently, acquisition success rates may be subject to reporting bias and should be interpreted with caution. Overall, this study primarily informs the design and operational planning of future prospective investigations evaluating the clinical utility of CEB in the prehospital setting.

## Data Availability

As the data generated in this study is of limited scope, it can be requested from the corresponding author on reasonable request.
